# Plant Growth Promotion Abilities of Phylogenetically Diverse *Mesorhizobium* Strains: Effect in the Root Colonization and Development of Tomato Seedlings

**DOI:** 10.3390/microorganisms8030412

**Published:** 2020-03-14

**Authors:** Esther Menéndez, Juan Pérez-Yépez, Mercedes Hernández, Ana Rodríguez-Pérez, Encarna Velázquez, Milagros León-Barrios

**Affiliations:** 1 Mediterranean Institute for Agriculture, Environment and Development (MED), Instituto de Investigação e Formação Avançada, Universidade de Évora, 7006-554 Évora, Portugal; esthermenendez@uevora.pt; 2 Departamento de Bioquímica, Microbiología, Biología Celular y Genética, Universidad de La Laguna, 38200 Tenerife, Canary Islands, Spain; juanernestoperezyepes@gmail.com (J.P.-Y.); anarguez@ull.es (A.R.-P.); mileonba@ull.edu.es (M.L.-B.); 3 Instituto de Productos Naturales y Agrobiología-CSIC, La Laguna, 38206 Tenerife, Canary Islands, Spain; mercedes@ipna.csic.es; 4 Departamento de Microbiología y Genética and Instituto Hispanoluso de Investigaciones Agrarias (CIALE), Universidad de Salamanca, 37007 Salamanca, Spain; 5 Unidad Asociada Grupo de Interacción Planta-Microorganismo, Universidad de Salamanca-IRNASA-CSIC), 37007 Salamanca, Spain

**Keywords:** *Mesorhizobium*, phylogeny, Canary Islands, plant root colonization, biofilms, plant growth promotion, tomato, biofertilization

## Abstract

*Mesorhizobium* contains species widely known as nitrogen-fixing bacteria with legumes, but their ability to promote the growth of non-legumes has been poorly studied. Here, we analyzed the production of indole acetic acid (IAA), siderophores and the solubilization of phosphate and potassium in a collection of 24 strains belonging to different *Mesorhizobium* species. All these strains produce IAA, 46% solubilized potassium, 33% solubilize phosphate and 17% produce siderophores. The highest production of IAA was found in the strains *Mesorhizobium* *ciceri* CCANP14 and *Mesorhizobium* *tamadayense* CCANP122, which were also able to solubilize potassium. Moreover, the strain CCANP14 showed the maximum phosphate solubilization index, and the strain CCANP122 was able to produce siderophores. These two strains were able to produce cellulases and cellulose and to originate biofilms in abiotic surfaces and tomato root surface. Tomato seedlings responded positively to the inoculation with these two strains, showing significantly higher plant growth traits than uninoculated seedlings. This is the first report about the potential of different *Mesorhizobium* species to promote the growth of a vegetable. Considering their use as safe for humans, animals and plants, they are an environmentally friendly alternative to chemical fertilizers for non-legume crops in the framework of sustainable agriculture.

## 1. Introduction

The plant growth promoting rhizobacteria (PGPR), also named plant probiotic bacteria, are promising biofertilizers for sustainable and environmentally friendly agriculture since they allow the total or partial substitution of chemical fertilizers added to the crops alone or together with organic amendments [1,2,3]. These bacteria take part in the plant microbiome and can live in the rhizosphere or endosphere of plants [4,5]. The bacteria inhabiting the inner plant tissues have advantages for plant interactions over the rhizospheric bacteria, including the promotion of plant growth [6]. Diverse mechanisms to promote plant growth are presented by plant probiotic bacteria, which involve direct and indirect effects. The direct mechanisms include the synthesis of phytohormones, such as the auxin indole-3-acetic acid (IAA), the uptake of essential nutrients through nitrogen fixation and solubilization of several insoluble compounds, such as phosphate, and the production of siderophores, which provides Fe to plants [7]. More recently, research has focused on potassium solubilization by different microorganisms; some of them are able to promote plant growth [8]. Within the indirect mechanisms are included the synthesis of antibiotics, lytic enzymes or siderophores involved in the control of plant pathogens [7,9].

Most of these mechanisms have been reported for rhizobia belonging to different genera and families [10,11,12]. In addition to their ability to fix atmospheric nitrogen with legumes [13], the production of the auxin IAA is probably the most common direct mechanism observed in strains from genera *Rhizobium* [14,15,16], *Phyllobacterium* [17] and *Mesorhizobium* [18,19], which can also synthesize and secrete siderophores [14,15,16,19]. Phosphate solubilization is a mechanism also widespread among PGP strains of *Rhizobium* [15,16], *Phyllobacterium* [17,20] and *Mesorhizobium* [19,21,22,23]; nevertheless, the ability to solubilize potassium has been reported to date only for one *Mesorhizobium* strain [24]. Therefore, the first aim of this study was to analyze all these plant promotion mechanisms (PGP) presented by several *Mesorhizobium* strains isolated from nodules of *Cicer canariense* in the Canary Islands that belonged to different bacterial species.

In addition to having several *in vitro* plant growth promotion mechanisms, good PGPR must be able to colonize the plant roots because this is an essential step for growth promotion [25]. The ability to colonize the roots of different non-leguminous plants has been shown for *Rhizobium* strains in tomato and pepper [14], strawberry [26], lettuce and carrots [15] and spinach [16], for a *Phyllobacterium* strain in strawberry [17] and for *Mesorhizobium* strains in lettuce and carrots [27]. Within these vegetables, the tomato (*Solanum lycopersicon* L.) is highlighted, whose production exceeds that of the other mentioned vegetables worldwide (http://www.fao.org/faostat/en/#home). The colonization of tomato roots by strains of *Bacillus* and *Paenibacillus* [28], *Rhizobium* [14], *Burkholderia* [29] and *Azospirillum* [30] has been reported; however, to date, there are no data for *Mesorhizobium* strains. Therefore, the second aim of this work was to analyze the ability of selected *Mesorhizobium* strains that have different *in vitro* plant growth–promotion mechanisms to colonize the tomato roots.

Recently, it has been shown that the formation of biofilms is essential to colonize the tomato roots with *Bacillus* strains. which can also form biofilms on abiotic surfaces [31]. The formation of biofilms in plant roots and abiotic surfaces has also been reported for *Rhizobium* strains able to nodulate different legumes [32] and for *Rhizobium* and *Phyllobacterium* able to promote the growth of strawberry and spinach plants [16,26]. These strains produce cellulose and cellulases, which are involved in biofilm formation [32] and developed microcolonies typical of biofilm initiation in the roots of strawberry [26] and spinach plants [16]. In the case of *Mesorhizobium,* the production of cellulases and cellulose has been shown for the strain ATCC 33669^T^ [32,33], which is currently the type strain of *Mesorhizobium jarvisii* [34]. However, there are no data about the production of biofilms in biotic and/or abiotic surfaces by any strain of genus *Mesorhizobium*. Therefore, the third aim of this study was to analyze the ability of the selected *Mesorhizobium* strains to produce cellulases and/or cellulose and to form biofilms in abiotic surfaces and tomato roots.

The strains actively colonizing the roots of plants can have a positive effect on their development. In the case of tomato, many researchers have analyzed the effect of the inoculation in a wide array of diverse bacteria, such as *Pseudomonas* [35,36,37,38], *Methylobacterium* [39], *Bacillus*, *Burkholderia* and *Pseudomonas* [40], *Azotobacter*, *Bacillus*, *Pseudomonas* and *Serratia* [41], *Paenibacillus* and *Bacillus* [28], *Rhizobium* [14], *Bacillus*, *Erwinia* and *Pseudomonas* [42], *Bacillus* [43], *Burkholderia* [29], *Alcaligenes* and *Bacillus* [44], *Streptomyces* [45], *Pseudomonas*, *Staphylococcus*, *Bacillus* and *Pantoea* [46], *Bacillus* [47], *Arthrobacter* and *Pseudomonas* [48] and *Bacillus* and *Acinetobacter* [49]. Nevertheless, there are no data about the effect of strains from the genus *Mesorhizobium* on tomato seedlings. Therefore, the final aim of this study was to analyze the effect of the inoculation of two selected *Mesorhizobium* strains showing *in vitro* PGP-activities on the growth of tomato seedlings. The results obtained showed for the first time that, similar to those of *Rhizobium*, *Mesorhizobium* strains are promising biostimulants for tomato plants.

## 2. Materials and Methods

### 2.1. Bacterial Strains

The rhizobia used in this study were isolated from effective root nodules of *C. canariense* from the Canary Islands in a previous work [50].

### 2.2. Phylogenetic Analysis

In this work, we performed the phylogenetic analysis of the *atpD* gene, amplified and sequenced using the primers and conditions previously described [51]. These sequences and those of the type strains of *Mesorhizobium* species described to date were aligned by using ClustalW software [52]. Distances calculated according to Kimura’s two-parameter model [53] were used to infer phylogenetic trees with the Neighbor-joining method [54] with MEGA7 software [55]. Confidence values for nodes in the trees were generated by bootstrap analysis using 1000 permutations of the data sets. The *atpD* sequences were deposited in the GenBank database under the accession numbers MN999428-MN999451.

### 2.3. Analysis of In Vitro Plant Growth Promoting (PGP) Mechanisms

For indole-acetic acid production, bacterial cultures were grown at 28 °C in YMB medium [56] supplemented with 2.5 mM L-tryptophan until reaching a stationary phase. Cells were eliminated by centrifugation and the IAA and IAA-like compounds were measured in the supernatants using a colorimetric method [57]. The phosphate-solubilizing ability was tested by growing the bacteria for 7–9 days in NBRIP medium [58] containing tricalcium phosphate as a source of insoluble phosphate and observing the formation of a transparent halo around the colony. Phosphate-solubilizing effectiveness was calculated as the ratio between the halos around the colony with respect to colony size [59]. Siderophore synthesis was evaluated by growing the bacteria for seven days in Chrome Azurol S medium (CAS)-agar medium [60], in which siderophore-producing strains had a yellowish halo around the colony [61]. The ability to solubilize potassium was tested using the Aleksandrov medium [62], which contains potassium aluminum silicate as K source. The presence/absence (+/−) of the halo was recorded at 14 days. 

### 2.4. Tomato Root Colonization and Biofilm Production Assays

Strains CCANP14 and CCANP122 were labeled with the green fluorescent protein (GFP) following a previously described protocol [63]. Tomato seeds were surface disinfected with 70% ethanol for 30 s followed by 5 min in 50% diluted commercial bleach. After six washes with sterile-distilled water, seeds were spread on 0.75% agar plates. Two days after germination, seedlings were transferred to 1.5% agar 10 cm × 10 cm square plates and each seedling was inoculated with 250 μL of a bacterial suspension (0.5 of OD_600_; 4 × 10^8^ CFU mL^−1^) and incubated in a growth chamber (16 h-light/8 h-dark cycle). Mock-inoculated controls were also included. Seedlings were viewed under a confocal microscope (Leica TCS SPE) five days after inoculation. Propidium iodide (7.5 µM) was used to counterstain plant root cells. The ability of strains CCANP14 and CCANP122 to form biofilms in abiotic surfaces was measured using the method of microtiter plate assay with crystal violet post-staining, following the protocol described by Robledo et al. [32]. The strains were grown in the minimal medium [32] and measurements were taken at 24, 48 and 72 hours. Biofilm data were treated with one-way ANOVA and the Tukey’s post hoc test at *p* ≤ 0.05, using RStudio version 1.1.463. Cellulase production was tested onto CMC double-layer plates as described previously [64] and the presence/absence of the halo was recorded at 7 days. Cellulose detection assays were performed as described by Robledo et al. [32].

### 2.5. Microcosm Plant Assays

For these assays, tomato (*Solanum lycopersicum* var. cherry) seeds were surface disinfected with 70% ethanol for 30 s followed by diluted commercial bleach (2.5% sodium hypochlorite) for 5 min. After six washes with distilled water, seeds were placed on 1% agar plates. Four days after germination, seedlings were planted in (12 cm × 12 cm) plastic pots containing 500 g of sterilized peat (Pro-line green peat, Compo, Münster, Germany). Sixteen plants per treatment were inoculated with the strains CCANP14 and CCANP122, independently, with 1 mL of bacterial cultures (five units in the McFarland standard, 1.5 × 10^9^ CFU·mL^−1^). As a control, a set of uninoculated plants were grown in the same conditions. Plants were irrigated with water every two days once a week with a nutrient solution [65] supplemented with 0.4 g·L^−1^ KNO_3_. Tomato plants were grown in a plant growth chamber with an 8 h-light/16 h-dark cycle. Five weeks after inoculation, the plants were harvested, roots washed with distilled water and fresh and dry weight (70 °C in an oven until constant weight was reached) of tomato shoots and roots were measured. For dry-weight measurement, the samples were dried in an oven at 80 °C. The dry plants were used for ionomic analyses, which were performed by the Ionomic service at IPNA (CSIC) using an ICP-OES AVIO500, Perkin Elmer equipment. Prior to analysis, the obtained data were checked for normality (Shapiro-Wilk test) and for homogeneity of variances (Levene’s test), and then, they were subjected to one-way ANOVA, using Fisher’s test (*p* = 0.05) by SPSS (version 21.0) statistical software (IBM, Chicago, IL, USA).

## 3. Results

### 3.1. Phylogenetic Analysis of the atpD Gene

**Figure 1:**
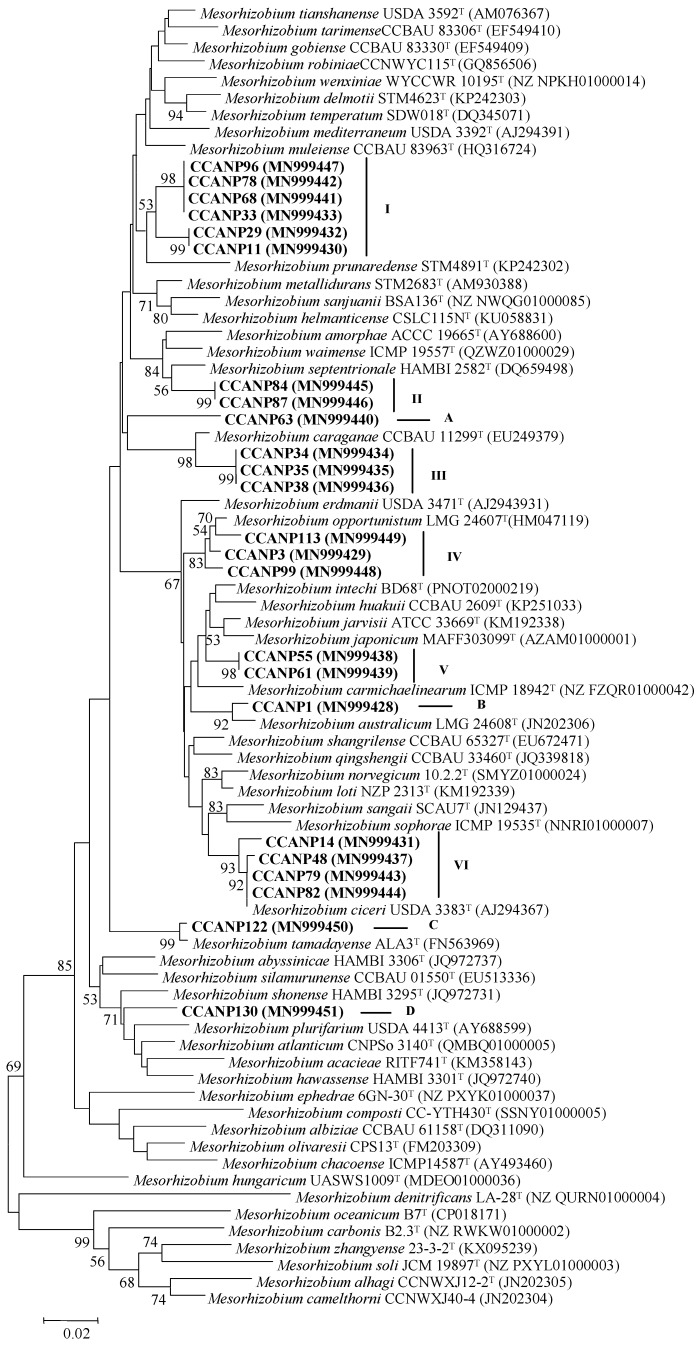
Neighbor-joining phylogenetic tree based on partial *atpD* gene sequences (870 nt) showing the position of the strains from this study within the genus *Mesorhizobium*. Bootstrap values calculated for 1000 replications are indicated. Bar, 2 nt substitutions per 100 nt. Accession numbers from GenBank are given in brackets.

In this work, we analyzed the *atpD* gene of 24 *Mesorhizobium* strains isolated in the Canary Islands from nodules of *C. canariense*, because this gene, which was not sequenced in our previous study [61], is commonly used for the differentiation of *Mesorhizobium* species. Furthermore, it is available for three recently described species, *Mesorhizobium denitrificans*, *Mesorhizobium carbonis* and *Mesorhizobium zhangyense*, for which the *glnII* gene, commonly included in the identification schemes of *Mesorhizobium* strains, is not available. The phylogenetic analysis of this gene showed that the analyzed strains belong to six clusters (I to VI) and four lineages (A to D) (Figure 1). Some of them can be confirmed as belonging to already described species (as they showed around 99% similarity values), namely, CCANP14, CCANP48, CCANP79 and CCANP82 to *M. ciceri*, CCANP3, CCANP99 and CCANP113 to *M. opportunistum*, CCANP1 to *M. australicum* and CCANP122 to *M. tamadayense* (Figure 1 and Table 1). The remaining strains were phylogenetically related to several *Mesorhizobium* species, but the similarity values were equal to or lower than 98% (Table 1). The strains CCANP11, CCANP29, CCANP33, CCANP68, CCANP78 and CCANP96 were closely related to *M. muleiense* with similarity values equal to or lower than 97%. The strains CCANP84 and CCANP87 have *M. septentrionale* as the closest relatives with 97.7% similarity value. The strains CCANP34, CCANP35 and CCANP38 show similarity values near to 98% to *M. caraganae*. The strains CCANP55 and CCANP61 were closest related to *M. jarvisii* with 96.6% similarity. Finally, the strains CCANP63 and CCANP130 formed independent phylogenetic lineages having 95.4% and 96.4% similarity, respectively, with respect to their closest related species *M. robiniae* and *M. shonense*.

### 3.2. In Vitro PGP Mechanisms

**Figure 2:**
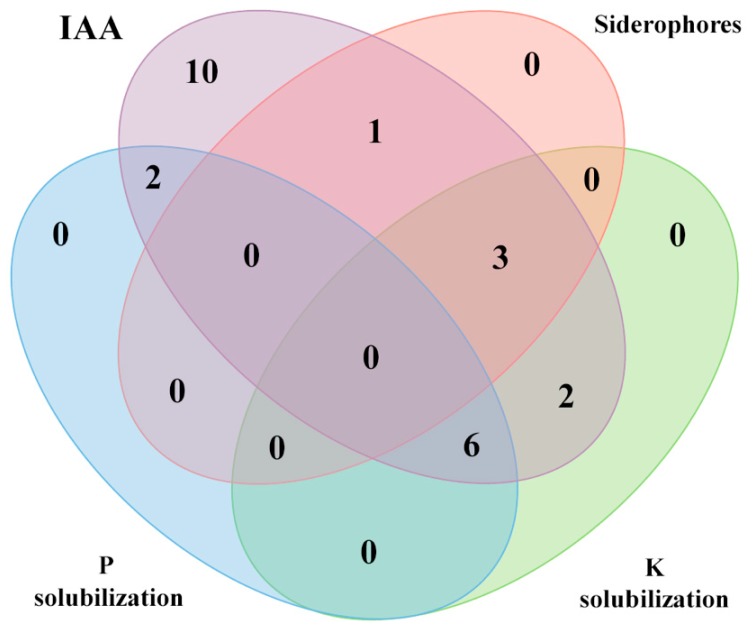
Four-set Venn diagram showing the number of the *Mesorhizobium* strains that have one or various of the following plant growth promotion mechanisms: IAA and siderophore production and K and P solubilization.

We have analyzed four of the most commonly PGP properties found in rhizobia, namely, IAA and siderophore production and phosphate and potassium solubilization, in the 24 *Mesorhizobium* strains from this study. The obtained results showed that they had at least one of these mechanisms (Figure 2). The IAA production was the most widespread trait of all strains synthesized in this auxin, although they varied greatly in the produced amounts from 5 to 69 µg mL^−1^ (Table 1). None of the strains possessed the four tested mechanisms; however, nine strains belonging to *M. ciceri*, *M. tamayadense* and *M. australicum* displayed three of them (Figure 2 and Table 1). Solubilization of tricalcium phosphate was detected in the four strains of *M. ciceri*, one strain of *M. opportunistum* and three strains related to *M. muleiense* (33%). Solubilization of potassium was detected in the four strains of *M. ciceri*, two strains of *M. opportunistum*, one strain of *M. australicum*, the strain of *M. tamadayense*, one strain related with *M. shonense* and three strains related to *M. muleiense* (46%). Both phosphate and potassium solubilization showed the highest indexes for the strains of species *M. ciceri* (Figure 2 and Table 1). The less frequent PGP trait in *Mesorhizobium* is the siderophore production, which was positive only in four strains (17%), two of them belonging to *M. australicum* and *M. tamadayense* and the other two strains related to *M. jarvisii* and *M. shonense* (Figure 2 and Table 1).

### 3.3. In vitro Biofilm Formation and Tomato Root Colonization

**Figure 3:**
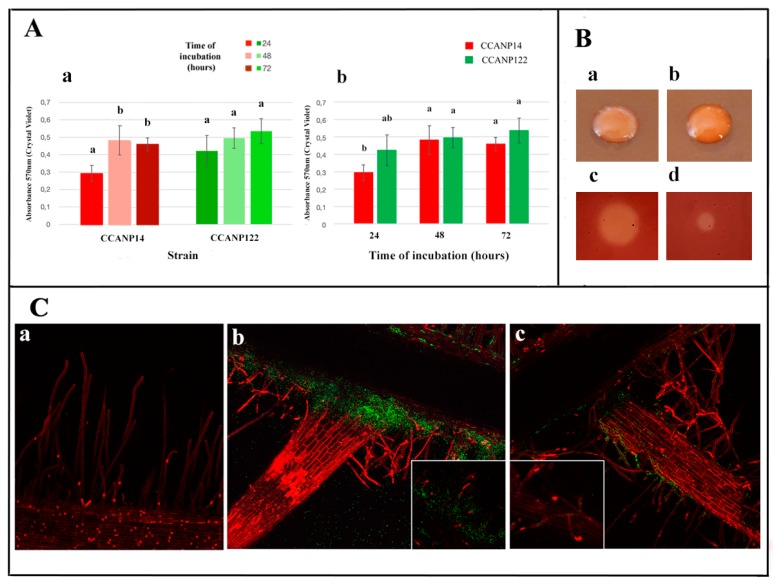
Panel (**A**) shows the absorbance values at OD_570_ of CV-stained biofilms formed on PVC plates by strains CCANP14 and CCANP122 at different incubation times (a) and evolution along the time of these values (b). Each graph bar represents the average of at least six wells. Error bars indicate the standard deviation. Values followed by the same letter do not differ significantly according to Tukey’s pos hoc test at *p* ≤ 0.05. Panel (**B**) shows the production of cellulose-like polysaccharides in Congo Red containing plates by the strains CCANP14 (a) and CCANP122 (b) and cellulase production on CMC (carboxy-methyl-cellulose) by the strains CCANP14 (c) and CCANP122 (d). Panel (**C**) shows tomato roots stained with propidium iodide as a negative control (a) and inoculated with GFP-tagged strains CCANP14 (b) and CCANP122 (c) observed with confocal laser scanning microscopy which showed the bacterial colonization at five days post-inoculation. Bars: 100 µm (a) and 200 µm (b,c) (50 µm in squared panels in (b and c)).

The biofilm formation, as we previously mentioned, is essential to colonize the plant roots, a process that also involves bacterial cellulose and cellulases. For the analysis of biofilm formation and cellulose and cellulase production, we selected two strains, *M. ciceri* CCANP14 and *M. tianshanense* CCANP122, because both strains produced the highest amounts of IAA; 68 μg mL^−1^ and 69 μg mL^−1^, respectively. Furthermore, CCANP14 showed the highest phosphate solubilization index and a high potassium solubilization index; CCANP122 also solubilizes potassium and produces siderophores. The in vitro biofilm formation assay revealed that the two *Mesorhizobium* strains are able to form biofilms (Figure 3A). An increase in the biofilm formation along the time was also found for both strains with significant differences only between the biofilm formation after 24 and 48 h for the strain CCANP14 (Figure 3Ab). Despite the lack of statistical significance, CCANP122 appeared to exhibit better biofilm formation than CCANP14, particularly at 24 h (Figure 3Aa). Both strains were able to produce cellulose in media containing Congo Red (Figure 3 Ba and Bb) and to produce cellulases, although the strain CCANP14 showed the highest activity (Figure 3 Bc and Bd).

Confocal scanning laser microscopy (CSLC) of GFP-tagged mesorhizobial strains showed that the two strains can colonize cherry tomato roots to different degrees and formed the typical three-dimensional biofilm structure (Figure 3C). Cherry tomato roots inoculated with strain CCANP14 displayed mature biofilms on the entire roots and root hairs (Figure 3Cb) five days post-inoculation, while CCANP122 (Figure 3Cc) colonized roots and root hairs in a more discrete manner.

### 3.4. Microcosm Plant Assays

The obtained results of the tomato inoculation with the strains CCANP14 and CCANP122 are shown in Table 2. Fresh and dry shoot and root lengths and weights of the inoculated plants were significantly higher than those of the uninoculated ones. Additionally, significant differences were found between the two inoculation treatments, except in the shoot length. The inoculation with CCANP14 produced the highest values in the remaining parameters (Table 2). Therefore, the inoculation of tomato seedlings with either of these two bacteria had a positive effect on plant growth, although the strain CCANP14 yielded the best results. 

The inoculation has an insignificant effect on the N and P content of plants (Table 3). Nevertheless, a significantly higher content of Ca was found in the shoots of plants inoculated with CCANP14 and the content of K and Na were significantly higher in those inoculated with CCANP122. The Zn content was significantly lower in the inoculated plants with respect to the control plants and the Mn content was significantly lower in the plants inoculated with the strain CCANP122 with respect to the control plants. The Fe content was significantly different between the plants from the two inoculation treatments and lower than in the control plants (Table 3).

## 4. Discussion

The strains analyzed in this study were previously distributed into 12 phylogenetic groups after the analysis of the housekeeping genes *dnaK*, *gyrB*, *truA*, *glnII*, *thrA*, *recA* and *rpoB*. The strains from some of these groups were identified as *M. australicum, M. ciceri*, *M. opportunistum* and *M. tamadayense*, but most of them belonged to an undescribed species of genus *Mesorhizobium* [66]. From these seven housekeeping genes, *glnII* and *recA* have been traditionally used for *Mesorhizobium* species differentiation and the description of new species. Nevertheless, for some recently described species of this genus, despite their genomes having been sequenced, the *glnII* gene is not available, whereas the *atpD* gene sequences are available in GenBank.

Therefore, the *atpD* gene sequences allowed us to compare our mesorhizobial strains with all described species within the genus *Mesorhizobium*. The results obtained in the present work showed that the *atpD* gene phylogeny was overall congruent with that obtained after the analysis of other protein-coding genes [66]. Thus, the *atpD* gene phylogeny confirmed the previous identification of the strains belonging to *M. australicum, M. ciceri, M. opportunistum* and *M. tamadayense* (clusters IV and VI and lineages B and C) (Figure 1) [66] as well as the phylogenetic location of strains within the clusters of *M. caraganae* (cluster III) and *M. septentrionale* (cluster II) (Figure 1) [66]. However, the phylogenetic position of some strains has changed because more than 10 *Mesorhizobium* novel species have been described since the publication of our previous work in the year 2014 [66]. This occurred with the strains CCANP55 and CCANP61 (cluster V), which had *M. huakuii* as their closest relative [66], whereas we show that currently the closest species is *M. jarvisii* [34] and confirm that the strains within this cluster V belong to a new *Mesorhizobium* lineage (Table 1). The strains CCANP11, CCANP29, CCANP33, CCANP68, CCANP78 and CCANP96 (cluster I) belong to a very divergent cluster that was originally defined as a *M. tianshanense*-like group [66]. Moreover, according to the *atpD* gene, their closest related species is *M. muleiense* (Table 1), which is included within a big clade of species with *M. tianshanense* (Figure 1). The strain CCANP63 (lineage A) formed an independent lineage related to *M. caraganae* [66] and has *atpD* gene sequence more similar to *M. robiniae* (Table 1), (Figure 1). Finally, the strain CCANP130 (lineage D) formed an independent lineage in our previous work [66] as well as in the *atpD* gene phylogeny, although the sequence of this gene was more similar to that of *M. shonense* (Table 1). Therefore, the results of the *atpD* gene analysis are congruent with those previously obtained after the analysis of other protein-coding genes [66].

Noteworthy is that the phylogenetic distances in the sequences of some housekeeping genes for several recently described *Mesorhizobium* species are lower than those found in older described species, as occurred for example for *Mesorhizobium japonicum* and *M. jarvisii*, whose classification into different species was mainly supported by the DNA-DNA relatedness values found between them [67]. Taking these two species as reference (Figure 1), the strains from clusters I, II, III and V and from lineages A and D belong to putative new species of genus *Mesorhizobium*, confirming the high taxonomic diversity of the *Mesorhizobium* strains occupying the *Cicer canariense* nodules (Table 1).

The capacity of species from genus *Mesorhizobium* to fix atmospheric nitrogen in symbiosis with legumes is widely known [68]; nevertheless, the presence of other *in vitro* plant growth promoting mechanisms has been less studied [12,19]. Among these mechanisms, IAA production is one of the most widespread PGP traits among *Mesorhizobium* strains [19] and this finding has been confirmed in this study, where the levels of IAA produced by some strains, particularly *M. ciceri* CCANP14 and *M. tamadayense* CCANP122 are similar to those found in *Rhizobium* strains able to promote the tomato growth [14]. The phosphate solubilization of tricalcium phosphate was one of the first plant growth promotion mechanisms reported for genus *Mesorhizobium*, specifically for a strain nodulating *Cicer arietinum* [69]. Later, it was confirmed that *Mesorhizobium* strains nodulating this legume were effective phosphate solubilizers [19,21], which is in agreement with the results from this study, where we found that strains related to *M. muleiense* and those of *M. ciceri*, two species originally isolated from *C. arietinum* nodules, showed the highest phosphate solubilization indexes (Table 1). The solubilization of potassium has only been reported to date for one strain of genus *Mesorhizobium* closely related to *Mesorhizobium plurifarium* isolated from rape rhizospheric soil [24]; now, this is the first report about the capacity to solubilize potassium of strains from different *Mesorhizobium* species isolated from legume nodules. The siderophore production was reported for some strains nodulating *C. arietinum* in the last years of the past decade [70] and later, Brígido et al. [19] reported this mechanism for several Portuguese strains nodulating this legume. In our work, siderophore production was detected in only four strains (Table 1). Therefore, it might be concluded that IAA production is a common PGP mechanism in *Mesorhizobium* strains, whereas phosphate solubilization and siderophore production are variable in agreement with the results of Brígido et al. [19]. These results are in agreement not only with those found for *Mesorhizobium* strains, but also for other rhizobia [14].

Some of these rhizobia can colonize the roots of non-legumes, as occurs with two *Rhizobium* strains that can colonize, amongst others, the roots of tomato [14]. In the case of genus *Mesorhizobium*, the root colonization of *Arabidopsis thaliana*, *Daucus carota* (carrots) and *Lactuca sativa* (lettuce) by two strains isolated from *Lotus* has been reported [27,71]; nevertheless, this is the first report about the ability of *Mesorhizobium* strains to colonize tomato roots. Root colonization is an essential step for plant growth promotion, and commonly, the better bacterial growth promoters also are good root colonizers, as occurs with several strains assayed on tomato plants, such as *Paenibacillus* and *Bacillus* [28], *Rhizobium* [14] and *Burkholderia* [29]. 

Some plant growth promoting strains are particularly effective in the promotion of tomato seedlings, whose production is carried out in nurseries exclusively dedicated to the commercialization of different seedlings, representing one of the most important economic resources in the agronomic field. For this reason, in many works the effect of different plant growth promoting bacteria on tomato seedlings have been evaluated, such as *Burkholderia* [40], *Azotobacter* and *Serratia* [41], *Rhizobium* [14], *Bacillus* [40,41,43,47], *Pseudomonas* [37,38,40,41] and *Arthrobacter* [48]. Nevertheless, this is the first study about the effect of *Mesorhizobium* strains on the growth and development of tomato seedlings. The obtained results showed that both of the assayed strains significantly improve the growth of shoots and roots of tomato seedlings without significantly affecting the percentages of the main macronutrients, N and P. This increase can be due to the biostimulant effect mediated by the IAA produced by these strains, as was reported for other bacteria producing this phytohormone [40]. Moreover, the significant increase of Ca is remarkable in seedlings inoculated with the strain CCANP14 because tomato plants have high requirements for this element involved in nutrition and tomato resistance to bacterial wilt diseases [72]. Additionally, there is a notable increase of K in seedlings inoculated with the strain CCANP122 since this element is involved in plant water regulation [73]. In the case of Mn and Zn, although their content was lower in inoculated plants, the values were higher than those considered enough for a suitable growth of tomato plants [74]. Although the ability to promote barley by a strain of *Mesorhizobium mediterraneum* [69] and Indian mustard by other strains of *Mesorhizobium loti* [75] had been previously reported, this is the first report about the ability of strains from different *Mesorhizobium* strains to promote the growth of the seedlings of a vegetable widely cultivated worldwide.

## 5. Conclusions

The results from this study showed that strains of different *Mesorhizobium* species have several plant growth-promoting mechanisms in addition to symbiotic N fixation, including IAA and siderophores production and phosphate and potassium solubilization. Selected PGPR strains of *M. ciceri* and *M. tamadayense* produce cellulases and cellulose that are able to form biofilms in abiotic surfaces and in roots of tomato increasing the growth of the seedlings of this plant. This is the first report about the potential of different *Mesorhizobium* species to promote the growth of a vegetable and considering their safety for human, animal and plant health after decades of use as bioinoculants. They are environmentally-friendly alternatives to chemical fertilizers for non-legume crops in the framework of sustainable agriculture.

## Figures and Tables

**Table 1:** Characteristics of *Mesorhizobium* strains isolated from *Cicer canariense* root nodules analyzed in this study.

Strains	Closest Species	*atpD* Gene Similarity (%)	Cluster or Lineage	IAA (µg mL^−1^)	Phosphate Solubilization ^¥^	Potassium Solubilization ^¥^	Siderophore Production ^§^
CCANP 1	*M. australicum*	98.6	B	8	0	1.50	1
CCANP 3	*M. opportunistum*	99.2	IV	23	2.00	1.41	0
CCANP 99	*M. opportunistum*	98.7	IV	23	0	0	0
CCANP 113	*M. opportunistum*	98.9	IV	23	0	1.10	0
CCANP 11	*M. muleiense*	96.8	I	33	0	1.10	0
CCANP 29	*M. muleiense*	97.0	I	40	1.40	1.10	0
CCANP 33	*M. muleiense*	96.8	I	35	0	0	0
CCANP 68	*M. muleiense*	96.8	I	37	1.72	0	0
CCANP 78	*M. muleiense*	96.8	I	24	0	0	0
CCANP 96	*M. muleiense*	96.8	I	33	2.10	ng	0
CCANP 14	*M. ciceri*	99.1	VI	68	2.40	1.39	0
CCANP 48	*M. ciceri*	99.8	VI	35	2.25	1.82	0
CCANP 79	*M. ciceri*	100	VI	49	2.06	1.57	0
CCANP 82	*M. ciceri*	100	VI	42	1.58	1.49	0
CCANP 34	*M. caraganae*	97.9	III	10	0	0	0
CCANP 35	*M. caraganae*	98.0	III	8	0	0	0
CCANP 38	*M. caraganae*	98.0	III	6	0	0	0
CCANP 63	*M. robiniae*	95.4	A	5	0	0	0
CCANP 55	*M. jarvisii*	96.6	V	40	0	0	0
CCANP 61	*M. jarvisii*	96.6	V	36	0	0	1
CCANP 84	*M. septentrionale*	97.7	II	31	0	0	0
CCANP 87	*M. septentrionale*	97.7	II	35	0	0	0
CCANP 122	*M. tamadayense*	99.4	C	69	0	1.22	1
CCANP 130	*M. shonense*	96.4	D	53	0	1.44	1

^¥^: Solubilization index. No solubilization (≤1 mm), low solubilization (1–1.5 mm) medium solubilization (1.5–2 mm) and high solubilization (≥2 mm). ^§^: Siderophore production index. No activity (0 mm), 1 (>0 and ≤5 mm). ng: no growth.

**Table 2:** Effect of inoculation of *Mesorhizobium ciceri* strain CCANP14 and *Mesorhizobium tamadayense* strain CCANP122 on the vegetative parameters of tomato plants.

Treatments	SL (cm/Plant)	RL (cm/Plant)	SFW (g/Plant)	RFW (g/Plant)	SDW (g/Plant)	RDW (g/Plant)
Control	12.71 (±1.17) a	24.89 (±1.02) a	4.18 (±0.53) a	0.43 (±0.01) a	0.27 (±0.02) a	0.05 (±0.01) a
CCANP14	20.46 (±1.03) b	34.33 (±1.64) c	15.95 (±1.44) c	3.15 (±0.38) c	1.52 (±0.15) c	0.38 (±0.05) c
CCANP122	23.25 (±1.80) b	29.08 (±1.20) b	14.34 (±1.11) b	2.16 (±0.17) b	1.15 (±0.09) b	0.21 (±0.02) b

Values followed by the same letter in each treatment are no significantly different from each other at *p* = 0.05 according to Fisher’s Protected LSD (Least Significant Differences). The numbers in parentheses are standard deviations. SL and RL: Shoot and Root length, respectively. SFW and RFW: Shoot and Root Fresh Weight, respectively. SDW and RDW: Shoot and Root Dry Weight, respectively.

**Table 3:** Effect of inoculation of *Mesorhizobium ciceri* strain CCANP14 and *Mesorhizobium tamadayense* strain CCANP122 on the mineral content of tomato shoots.

Treatments	N (g kg^−1^)	P (g kg^−1^)	Ca (g kg^−1^)	K (g kg^−1^)	Mg (g kg^−1^)	Na (g kg^−1^)	Fe (mg kg^−1^)	Mn (mg kg^−1^)	Cu (mg kg^−1^)	Zn (mg kg^−1^)
Control	4.4 (±0.2) a	3.1 (±0.2) a	12.6 (±0.8) a	54.5 (±2.4) a	4.9 (±0.4) a	1.4 (±0.1) a	157.3 (±8.6) ab	45.8 (±2.5) a	7.8 (±0.5) a	66.0 (±2.8) a
CCANP14	3.9 (±0.1) a	3.1 (±0.2) a	18.4 (±1.8) b	58.1 (±1.6) a	5.4 (±0.4) a	1.3 (±0.1) a	195.8 (±3.6) a	38.0 (±2.5) ab	9.5 (±1.2) a	47.3 (±2.3) b
CCANP122	4.2 (±0.1) a	3.5 (±0.1) a	11.6 (±0.4) a	66.4 (±1.2) b	3.8 (±0.1) a	2.0 (±0.1) b	107.5 (±5.4) b	36.0 (±2.3) b	8.8 (±0.3) a	44.8 (±4.7) b

Values followed by the same letter in each treatment are no significantly different from each other at *p* = 0.05 according to Fisher’s Protected LSD (Least Significant Differences). Units are expressed in g kg^−1^. The numbers in parentheses are standard deviations.
